# Co‐Occurrence of Resistance to Aminoglycosides and Carbapenems in *Klebsiella pneumoniae*: A Critical Threat to Public Health

**DOI:** 10.1155/ijm/4503589

**Published:** 2026-04-26

**Authors:** Rafael Artur de Queiroz Cavalcanti de Sa, Tainara Fernandes Dantas, Sérgio Costa Júnior, Karolayne Oliveira da Silva, Maria Cecília Ferreira Galindo, Amanda Vieira de Barros, John Eversong Lucena de Vasconcelos, Radosław Kowalski, Grażyna Kowalska, Rafal Rowinski, Henrique Douglas Melo Coutinho, Maria Betânia Melo Oliveira

**Affiliations:** ^1^ Department of Biochemistry, Federal University of Pernambuco, Recife, Brazil, ufpe.br; ^2^ Department of Microbiology, University Center of Vitória de Santo Antão, Brazil; ^3^ Department of Dentistry, CECAPE College, Brazil; ^4^ Department of Analysis and Evaluation of Food Quality, University of Life Sciences in Lublin, Lublin, Poland, up.lublin.pl; ^5^ Department of Tourism and Recreation, University of Life Sciences in Lublin, Lublin, Poland, up.lublin.pl; ^6^ Department of Biological Chemistry, Regional University of Cariri, Crato, Ceara, Brazil, urca.br

**Keywords:** antibiotics, antimicrobial resistance, epidemiology, nosocomial infections

## Abstract

Antimicrobial resistance (AMR) in Klebsiella pneumoniae, especially the co‐occurrence of resistance to aminoglycosides and carbapenems, represents a serious threat to global public health. This study reviews the prevalence of this combined resistance, analyzes the underlying mechanisms, and evaluates its impact on clinical practice and public health. To this end, a narrative literature review was carried out, using databases such as PubMed, SciELO, Google Scholar, DOAJ, Virtual Health Library (VHL), and ScienceDirect. Studies published in the last 10 years in English and Portuguese, related to resistance to these two drugs in K. pneumoniae, were included. The qualitative analysis of the data revealed an alarming increase in the prevalence of resistant strains, mainly associated with the production of Aminoglycoside Modifying Enzymes (AME) and carbapenemases, resulting in significantly high rates of mortality, morbidity, and treatment costs. To mitigate this threat, multifaceted strategies are needed, such as the rational use of antibiotics, epidemiological surveillance, education programs, and investment in research for new antimicrobials. In addition, it is observed that multisectoral collaboration is essential to face this challenge, requiring coordinated and continuous efforts in research, education, and public health practices.

## 1. Introduction

Antimicrobial resistance (AMR) has been recognized as one of the main threats to global public health in the 21st century, characterized by the emergence and rapid spread of multidrug‐resistant (MDR) pathogens in both clinical and community environments [1]. Among these pathogens, *Klebsiella pneumoniae* stands out as a significant concern, due to its remarkable ability to develop and propagate resistance to multiple classes of antibiotics [2]. In 2024, the World Health Organization (WHO) classified this species among the critical priority pathogens, underlining the severity of the threat it poses [3].

Recent evidence highlights the emergence of hypervirulent *K. pneumoniae* (hvKP), characterized by capsular types (K1/K2), siderophores, and virulence plasmids. Reports of hvKP acquiring carbapenemase genes raise major clinical and epidemiological concerns, creating strains that combine high virulence with multidrug resistance and complicate treatment strategies [3–5].

Recent studies have shown a worrying increase in the prevalence of carbapenems and aminoglycoside (CAM‐KP) resistance in *K. pneumoniae*, both considered last‐choice options, essential for the treatment of severe infections caused by Gram‐negative bacteria [6]. The co‐occurrence of resistance to these classes of antibiotics severely compromises the available therapeutic options, resulting in adverse clinical outcomes that affect patients’ lives [7, 8].

The global spread of high‐risk clones such as ST258, ST11, ST147, and ST307 has been strongly associated with carbapenem and aminoglycoside resistance. These lineages frequently harbor *blaKPC*, *blaNDM*, or *blaOXA-48-like* genes and mobile genetic elements (MGEs), facilitating rapid dissemination and increasing outbreak potential [6, 9]).

Aminoglycosides resistance is largely mediated by AMEs, which inactivate antibiotics through adenylation, phosphorylation, or acetylation mechanisms. On the other hand, resistance to carbapenems is often associated with carbapenemase synthesis, which hydrolyzes the *β*‐lactam ring of these antibiotics. The most prevalent carbapenemases in clinical isolates of *K. pneumoniae* include KPC (*K. pneumoniae* carbapenemase), NDM (New Delhi metallo‐*β*‐lactamase), and OXA‐48 [7].

Additional mechanisms, such as porin loss (OmpK35/OmpK36 mutations) and efflux pump overexpression, further enhance resistance levels when combined with *β*‐lactamase production. These alterations, together with MGEs, contribute to the rapid spread of multidrug resistance [10, 11].

The co‐occurrence of resistance to these two categories of drugs in *K. pneumoniae* has significant consequences for public health. Infections associated with these multi‐resistant bacteria are correlated with high rates of mortality and morbidity, as well as substantial costs for health systems, resulting from prolonged hospital stays and more expensive alternative therapy needs [1, 12]. While global surveillance reports indicate rising carbapenem resistance in *K. pneumoniae*, Brazilian data reveal a concerning increase in *blaNDM* detection and persistently high ICU resistance rates, emphasizing the need for regionally adapted strategies integrated into global monitoring systems [13–15]. These trends underscore critical gaps in genomic surveillance and One Health approaches, reinforcing the importance of comprehensive reviews to guide clinical practice and public health interventions.

Given this alarming scenario, this study reviewed the prevalence of co‐occurrence of aminoglycoside and carbapenem resistance in strains of *K. pneumoniae*, in addition to analyzing the main resistance mechanisms involved and the impact of this resistance on clinical practice and public health. This review additionally discussed potential strategies to mitigate the spread of this resistance and improve therapeutic outcomes in an attempt to contribute to the preservation of the effectiveness of antimicrobial treatments for infections caused by *K. pneumoniae* and, consequently, assists in the protection of public health.

Furthermore, this review incorporates a genomic perspective, highlighting the role of whole‐genome sequencing (WGS) in outbreak investigation, high‐risk clone tracking, and detection of resistance determinants, as well as the importance of a One Health approach to address the interconnectedness of human, animal, and environmental reservoirs [16, 17].

## 2. Methodology

**Figure 1:**
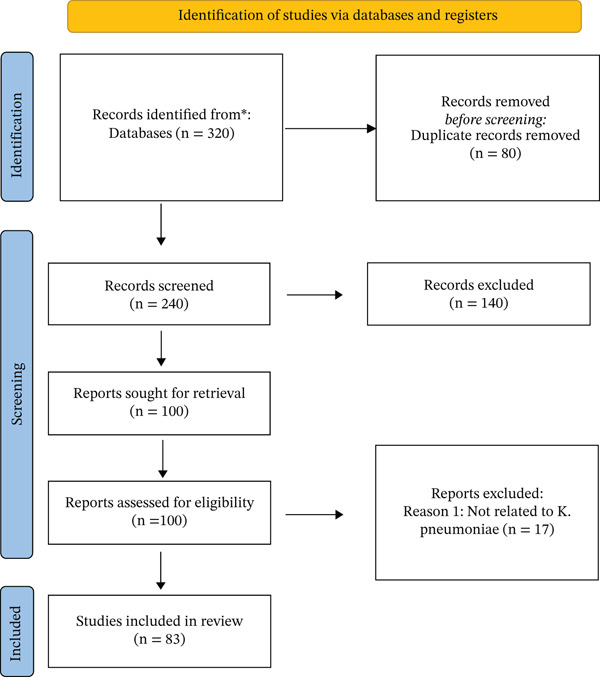
PRISMA flow diagram illustrating the study selection process.

This study was conducted as a narrative literature review aimed at synthesizing and discussing evidence on the co‐occurrence of aminoglycoside and carbapenem resistance in *K. pneumoniae*, emphasizing its public health impact. The narrative approach was chosen for its ability to provide an exploratory and comprehensive analysis, integrating findings from diverse methodologies and contexts, which is essential to understand the complexity of AMR. Although this study is a narrative review, a PRISMA‐style flow diagram (Figure 1) is included for illustrative purposes to ensure transparency in the selection process. The 83 studies were selected based on a basic screening of titles and abstracts, prioritizing those that provided primary data on co‐resistance prevalence or molecular mechanisms of *K. pneumoniae*. Quality was assessed by verifying the presence of clear microbiological methods and peer‐review status.

The review followed a PECO strategy to guide the search and selection process:

• Population (P): Clinical isolates of *K. pneumoniae*.
• Exposure (E): Resistance to aminoglycosides and carbapenems.
• Comparison (C): Susceptible strains or global versus Brazilian resistance patterns.
• Outcome (O): Clinical impact, epidemiology, and public health implications.

The bibliographic search was carried out between May and July 2024 in databases and electronic libraries including PubMed, SciELO, Google Scholar, DOAJ, Virtual Health Library (VHL), and ScienceDirect. Search terms combined MeSH/DeCS descriptors and Boolean operators:

*“Antimicrobial Resistance”* AND *“Klebsiella pneumoniae”* AND *“Aminoglycosides”* AND *“Carbapenems”* AND *“Beta-Lactamases”* AND *“Carbapenemases”* AND *“Multiresistance”* AND *“Clinical Impact”*.

Filters were applied for studies published between 2015 and 2024 in English or Portuguese. No protocol was registered for this review due to its narrative design.

Studies were included if they addressed resistance to aminoglycosides and carbapenems in *K. pneumoniae* and discussed clinical or public health implications. Review articles, observational studies, clinical trials, and reports from public health organizations were considered. Exclusion criteria comprised non‐peer‐reviewed studies, unrelated species, insufficient data, or inadequate methodological quality.

This review is limited by its narrative nature, potential publication bias, and heterogeneity of included studies. No meta‐analysis was performed due to variability in study designs and outcome measures.

## 3. Results and Discussion

This narrative review analyzed 83 studies to comprehensively evaluate the co‐occurrence of aminoglycoside and carbapenem resistance in *K. pneumoniae* and its implications for public health. The study selection process, summarized in Figure 1, identified 320 records from databases, with 83 studies meeting the inclusion criteria for qualitative synthesis. The findings reveal an alarming global prevalence of co‐resistance, with particularly critical rates in Latin America and Brazil, where carbapenem resistance exceeds 50% in ICU settings. The analysis identified that this epidemiological scenario is driven by a complex interplay of molecular mechanisms, including the production of aminoglycoside‐modifying enzymes (AMEs) and carbapenemases (KPC, NDM, OXA‐48), frequently associated with high‐risk clones (ST258, ST11, ST147, ST307) and facilitated by MGEs. The convergence of classical (cKP) and hypervirulent (hvKP) pathotypes further exacerbates the clinical challenge, combining multidrug resistance with enhanced virulence. These factors collectively contribute to increased mortality, prolonged hospitalization, and elevated treatment costs, underscoring the urgent need for integrated interventions spanning antimicrobial stewardship, robust surveillance, and innovative therapeutic approaches within a One Health framework.

### 3.1. Prevalence of Co‐Occurrence of Resistance in K. pneumoniae

#### 3.1.1. Epidemiological Data

Aminoglycosides and carbapenems resistance in *K. pneumoniae* is a growing concern worldwide, reflecting a significant challenge to global public health [18]. Epidemiological studies indicate that the prevalence of this resistance varies considerably between different regions, showing the influence of antibiotic use practices and infection control policies [6]. In developed countries, such as the United States and Western Europe, carbapenem resistance rates have increased alarmingly, with reports of resistance of more than 20% in some health institutions. In Latin America, resistance to these antibiotics can reach worrying levels, exceeding 40%, largely due to the selective pressure resulting from the excessive and inadequate use of antimicrobials [19].

This variation in resistance rates is also influenced by sociocultural and economic factors that affect the administration of antibiotics [20]. In developing countries, where access to quality health care is often limited, practices such as self‐medication and inadequate antibiotic prescription become common. These factors contribute to the selection of resistant strains, perpetuating a cycle of infections that are difficult to treat. Therefore, it is essential to understand the regional dynamics of resistance to implement effective control and prevention strategies [15].

In addition, the lack of systematic monitoring of AMR in many countries results in underreporting, making it difficult to obtain an accurate overview of the situation [15]. The collection of robust epidemiological data is vital to identify trends and evaluate the effectiveness of public health interventions. Global Surveillance initiatives, such as the WHO Global Antimicrobial Resistance Surveillance System, are crucial to improve the understanding of resistance and provide data that can inform more effective and targeted health policies [3].

Resistance to aminoglycosides and carbapenems in *K. pneumoniae* represents a critical global public health challenge. Our analyses reveal significant regional disparities: while global surveillance indicates carbapenem resistance rates of 8%–20% [15], Brazilian data from ANVISA [13] demonstrate alarming rates exceeding 50% in ICUs, with similar trends observed across Latin America [14]. This scenario reflects the persistence of high resistance rates in Brazilian ICUs between 2019 and 2024, particularly for carbapenems, following a period of stability in the combined phenotype (3rd/4th generation cephalosporins + carbapenems) observed from 2015 to 2018. This regional variation underscores the influence of local antibiotic stewardship practices and healthcare infrastructure on resistance dynamics, highlighting the urgent need for integrated strategies to preserve last‐line therapeutic options. A comparative summary of resistance rates is presented in Table 1.

**Table 1:** Comparative resistance rates of *Klebsiella pneumoniae* to carbapenems and aminoglycosides by region and source.

Region/Source	Imipenem (%)	Meropenem (%)	Amikacin (%)	Gentamicin (%)	Year/Source
**Brazil (ANVISA)**	61–67	61–67	10–12	44–47	2021–2024, Reports ANVISA
**Latin America (RELAVRA/PAHO)**	30–50+	30–50+	≤ 10–20	30–50	2022, RELAVRA
**Global (WHO-GLASS)**	8–20	8–20	< 10	20–30	2022, WHO‐GLASS
**Molecular studies (Brazil + LatAm)**	—	—	Genes: *rmtB, ArmA, RmtD* coexisting with *KPC-2/NDM-1*; Clones: ST258, ST11, ST147, ST307	Reported outbreaks with resistance up to 40%–50%	2012–2022, multicenter studies

*Note:* Resistance rates based on global and regional surveillance data. The “Molecular Studies” row highlights genes associated with combined resistance and high‐risk clones frequently implicated in hospital outbreaks. Source: Prepared by the authors based on literature data (2024).

#### 3.1.2. Clinical Environments

The analysis of resistance in *K. pneumoniae* in different clinical environments reveals significant differences between hospitals and communities [21, 22]. In hospitals, the selective pressure resulting from the frequent use of broad‐spectrum antibiotics leads to a higher incidence of resistant strains. Infections acquired in intensive care units, for example, have a high prevalence of resistance to aminoglycosides and carbapenems, with rates that often exceed 50% in some critical patient populations [6]. This resistance not only compromises treatment options, but is also associated with high rates of complications and mortality, highlighting the severity of the problem in hospital contexts [21, 22].

On the other hand, community infections caused by this species are relatively less frequent, although their incidence is on the rise. Studies indicate that, although resistance is less common outside the hospital environment, resistant strains are emerging in communities, especially among vulnerable populations, such as the elderly and immunocompromised individuals [21, 22]. The analysis of the most prevalent infections associated with resistant *K. pneumoniae*, such as urinary tract infections and pneumonia, emphasizes the need for continuous surveillance and preventive interventions, even in community environments [21, 22].

Given this scenario, it is crucial that the approach to the management of *K. pneumoniae* infections considers the differences between clinical environments. Specific strategies for infection control in hospitals, such as the implementation of strict hygiene protocols and active surveillance, are essential to limit the spread of resistant strains [3]. Simultaneously, the education of the population and the promotion of the responsible use of antibiotics in communities are equally important to prevent the emergence of resistance and protect public health [23].

#### 3.1.3. Resistance Contributing Factors

Antibiotic resistance in *K. pneumoniae* is influenced by a variety of factors, with the indiscriminate use of antimicrobials being one of the main contributors [20]. The inadequate administration of antibiotics, either by self‐medication or by the absence of strict clinical guidelines, results in a selective pressure that favors the survival of resistant strains. The excessive prescription of antibiotics in situations where they are not necessary, such as in viral infections, further aggravates this problem, leading to an increase in resistance in bacterial populations [23].

In addition to the use of antibiotics, factors related to the patient and the hospital environment play a significant role in resistance. Immunocompromised patients, for example, are more susceptible to resistant *K. pneumoniae* infections due to their reduced ability to fight infections [24]. The lack of adequate infection control practices in hospital environments, such as hand hygiene and surface disinfection, can facilitate the transmission of resistant strains between patients [25]. These conditions create an environment conducive to the spread of resistant infections, complicating clinical management and increasing the burden on health systems.

To face these contributing factors, it is essential to implement multifaceted strategies. This includes the promotion of the rational use of antibiotics, the education of health professionals on AMR and adherence to strict infection control protocols [26]. Awareness of the risks associated with the inappropriate use of antibiotics, accompanied by better practices, can significantly contribute to the containment of resistance in *K. pneumoniae* and to the improvement of clinical outcomes [19].

### 3.2. Resistance Mechanisms

#### 3.2.1. Aminoglycosides Resistance Mechanisms

Aminoglycosides remain a mainstay in the treatment of severe bacterial infections; however, *K. pneumoniae* has evolved sophisticated strategies to evade these agents [27]. The predominant resistance mechanism is the production of AMEs, which chemically inactivate the drugs through acetylation, phosphorylation, or nucleotidylation. Among the most clinically relevant AMEs found in *K. pneumoniae* are AAC(3)‐IV, APH(3 ^′^), and ANT(2 ^′^) [28]. These enzymes act by modifying specific positions on the aminoglycoside molecule, thereby preventing its successful binding to the bacterial ribosome and rendering the treatment ineffective [28].

Beyond individual enzymatic activity, the evolutionary success of these resistance traits is driven by their mobility. AME‐encoding genes are frequently carried on diverse MGEs, such as plasmids, integrons, and transposons, which facilitate rapid horizontal gene transfer across different *K. pneumoniae* lineages and even between distinct bacterial species [9, 11, 29]. This dissemination is particularly aggressive in hospital settings, where high selective pressure from intensive antibiotic use promotes the persistence and spread of these genetic elements, leading to a surge in MDR infections [9]. Consequently, a deep molecular understanding of these pathways, combined with robust genomic surveillance, is paramount for refining clinical guidelines and informing the design of next‐generation antimicrobials capable of bypassing enzymatic degradation.

#### 3.2.2. Carbapenem Resistance Mechanism

Carbapenems are a pivotal last‐line defense against resistant Gram‐negative pathogens; however, *K. pneumoniae* has developed an alarming capacity for resistance through the production of carbapenemases ([30]; BAHAR et al., 2020). These enzymes effectively hydrolyze the *β*‐lactam ring, neutralizing the antibiotic’s efficacy before it reaches its target. The genes encoding these enzymes are predominantly carried on MGEs, such as conjugative plasmids, facilitating rapid horizontal transfer and widespread dissemination across *K. pneumoniae* lineages and other Enterobacteriaceae [9, 11].

Among the most prevalent carbapenemases, *K. pneumoniae* carbapenemase (KPC), New Delhi metallo‐*β*‐lactamase (NDM), and OXA‐48‐like *β*‐lactamase (OXA‐48) are of significant clinical concern [27, 31]. While KPC remains the most frequent variant in hospital outbreaks globally, the distribution of NDM and OXA‐48 continues to expand, driven by their high stability and transfer efficiency via epidemic plasmids [6, 9, 11].

**Figure 2:**
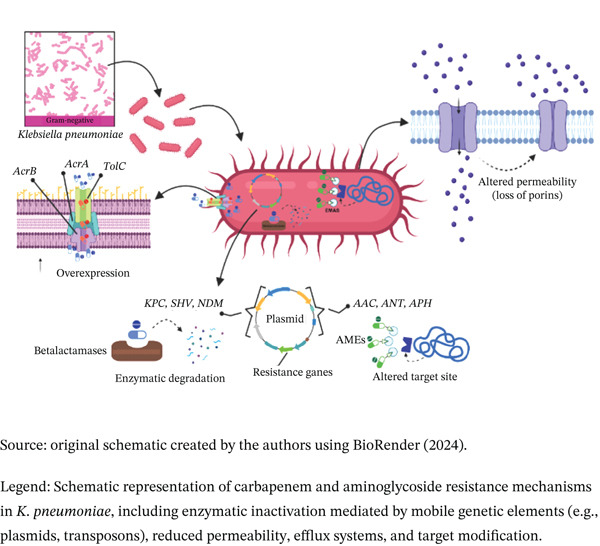
Molecular resistance mechanisms (carbapenem and aminoglycosides).

Beyond enzymatic hydrolysis, resistance is often exacerbated by reduced outer membrane permeability and active efflux. Mutations or decreased expression of major porins, particularly OmpK35 and OmpK36, significantly restrict antibiotic entry into the cell [30, 32]. Simultaneously, the overexpression of efflux pumps contributes to the active expulsion of the drug, further diminishing therapeutic efficacy [10]. As illustrated in Figure 2, this synergy between enzymatic destruction, impaired permeability, and active efflux creates a multifaceted resistance profile that severely limits contemporary treatment options.

#### 3.2.3. Pathotypic and Virulence Convergence in *K. pneumoniae*

Classical *K. pneumoniae* (cKP) remains predominantly associated with healthcare‐acquired infections, particularly pneumonia, urinary tract, and bloodstream infections in vulnerable inpatients. In contrast, hypervirulent *K. pneumoniae* (hvKP) has emerged as a distinct pathotype capable of causing invasive disease—including liver abscess, endophthalmitis, and meningitis—even in previously healthy hosts. hvKP typically harbors a thicker polysaccharide capsule (frequently K1/K2), an expanded siderophore repertoire (e.g., aerobactin/salmochelin), and regulators such as rmpA/rmpA2, which enhance capsule production and virulence [33–35].

A mounting concern is the convergence of virulence and resistance, whereby hvKP acquires carbapenemase genes (e.g., *bla*KPC, *bla*NDM) and 16S rRNA methyltransferases (e.g., *ArmA, RmtB*), generating highly virulent and extensively drug‐resistant lineages. These hv‐CRKP strains complicate therapy, heighten the risk of severe outcomes, and increase outbreak potential in both hospital and community interfaces. Their detection and containment require integration of molecular markers of virulence with resistance profiling in routine surveillance and outbreak investigations [21, 22, 27, 31].

Addressing the threat posed by convergent strains requires enhanced diagnostic approaches that simultaneously detect virulence and resistance markers. The implementation of molecular techniques, such as PCR for virulence genes (*rmpA*, *aerobactin*) alongside carbapenemase gene detection, can provide early warning of emerging threats. Furthermore, infection control measures must be adapted to account for the unique transmission dynamics of hvKP, including potential community spread. Ongoing surveillance programs should incorporate pathotype characterization to inform targeted interventions and antibiotic stewardship strategies tailored to these high‐risk lineages [1, 12].

#### 3.2.4. Interaction Between Resistance Mechanisms

The co‐occurrence of different resistance mechanisms in *K. pneumoniae* results in a multi‐resistant resistance phenomenon, which represents a significant challenge in clinical practice. When strains of this species simultaneously express AMEs and carbapenemases, the effectiveness of antimicrobial treatment is severely compromised. This interaction between resistance mechanisms can create a synergy that allows bacteria to become resilient to multiple classes of antibiotics, making it even more difficult to manage infections ([36]).

A remarkable example of this phenomenon is observed in *K. pneumoniae* strains that produce both KPC and AMEs. These strains are often isolated in hospital environments and have been associated with outbreaks of infections, especially in Intensive Care Units (ICU). The simultaneous presence of multiple resistance mechanisms not only increases the complexity of treatment but also increases the mortality and morbidity rates associated with these infections (BAHAR et al., 2020; [9]). Therefore, the identification and monitoring of these resistant strains are essential to develop effective treatment strategies.

Thus, it is observed that the interaction between the different mechanisms of resistance in *K. pneumoniae* represents a growing concern in the field of AMR. Understanding this complexity is essential to guide research efforts and develop interventions that can mitigate the spread of resistance. The investigation of new treatments and approaches to reverse resistance is extremely important to ensure that antimicrobial therapy remains an effective tool in the fight against bacterial infections [37, 38].

#### 3.2.5. High‐Risk Clones and Surveillance Implications

The global dissemination of carbapenem and aminoglycoside co‐resistance is largely driven by specific high‐risk clones. Sequence types ST258, ST11, ST147, and ST307 have been consistently associated with *blaKPC*, *blaNDM*, and OXA‐48‐like carbapenemases, often co‐carried with aminoglycoside resistance determinants (e.g., *armA*, *rmtB*) on conjugative plasmids (BAHAR et al., 2020; [6, 9]). A summary of the major high‐risk clones, their associated resistance genes, and geographical distribution is presented in Table 2. These epidemic lineages demonstrate remarkable capacity for international spread and have been implicated in numerous hospital outbreaks, particularly in critical care settings. The genetic plasticity of these clones, facilitated by integron and transposon systems, enables rapid acquisition and dissemination of resistance determinants across diverse geographical regions [21, 22, 24].

**Table 2:** High‐risk clones of *Klebsiella pneumoniae* associated with resistance.

Clone (ST)	Carbapenemase(s)	Genes of AMG resistance	Region	Clinical context	References
ST11	*blaKPC, blaND*M	*rmtD, armA*	Asia, Latin America	Regional epidemics	[24]; [11]; [31]
ST147	*blaNDM, blaOXA-48-like*	*rmtB, rmtD*	Europe, Latin America	Inter‐Hospital transmission	[27]; [9]; [15]
ST258	*blaKPC*	*armA, rmtB*	USA, Europe	Hospital outbreaks, ICU	[9]; [6]; Bahar et al., 2020; [21, 22]
ST307	*blaKPC*	*armA*	Latin America, USA	Emerging outbreaks	[21, 22]; [6]; [15]

*Note:* This table summarizes the major high‐risk clones of *Klebsiella pneumoniae* (sequence types, STs) associated with carbapenem and aminoglycoside resistance. It includes the most common carbapenemases, aminoglycoside resistance genes, predominant geographic regions, and key references supporting these associations. These clones are critical for global and regional surveillance due to their role in outbreaks and rapid dissemination of multidrug resistance. Source: Prepared by the authors based on literature data (2024).

In practical terms, WGS adds resolution to routine surveillance by linking patient‐to‐patient transmission, identifying outbreak clusters, and mapping resistance/virulence cassettes across plasmid backbones and integrons/transposons. The incorporation of WGS data into national systems (e.g., alignment with GLASS indicators) strengthens early warning for the expansion of high‐risk clones and supports targeted interventions (cohorting, de‐escalation, and stewardship focusing on last‐line agents). In Latin America and Brazil, reports of *blaNDM* rise and ICU‐level resistance further justify a WGS‐enabled network integrated with conventional phenotyping to curb the spread of ST11/ST147/ST307 lineages [11, 15, 24].

However, despite WGS being the gold standard for high‐resolution tracking, its implementation in low‐ and middle‐income countries (LMICs) faces significant practical hurdles. Socioeconomic factors and limited access to quality healthcare significantly affect the management of AMR in developing nations [20]. Furthermore, the lack of systematic monitoring and underreporting, as highlighted by global surveillance reports, obscures the true burden of bacterial resistance in these regions [15]. In the specific context of Brazil and Latin America, high operational costs and inadequate laboratory infrastructure remain critical barriers to large‐scale genomic surveillance [11, 24]. Therefore, public health strategies in these settings should prioritize a hybrid approach, combining cost‐effective molecular screening with centralized genomic surveillance to ensure effective clinical decision‐making.

Furthermore, the adoption of emerging surveillance technologies, such as real‐time long‐read sequencing and wastewater‐based genomic epidemiology, represents the next frontier in AMR monitoring. Recent studies have demonstrated that portable long‐read sequencing platforms (e.g., Oxford Nanopore) enable rapid and cost‐effective AMR surveillance even in resource‐limited settings [39]. Additionally, the implementation of wastewater‐based epidemiology using long‐read metagenomic sequencing has proven effective for tracking resistance trends in the community before they reach clinical environments [40], providing a more proactive and integrated public health response.

To effectively contain the spread of high‐risk clones, a multi‐pronged approach is essential. This includes strengthening international collaboration for real‐time data sharing on emerging clones, implementing cost‐effective WGS in reference laboratories, and developing rapid molecular assays for frontline detection of epidemic lineages. Additionally, infection control teams should establish clone‐specific containment protocols, and antimicrobial stewardship programs must be optimized to address the resistance profiles prevalent in circulating high‐risk clones. These coordinated efforts are crucial for mitigating the public health impact of these successful epidemic lineages [2, 19].

### 3.3. Impact on Clinical Practice

#### 3.3.1. Clinical Outcomes

Infections caused by resistant *K. pneumoniae* are associated with significantly higher mortality and morbidity rates compared with infections caused by sensitive strains. Research shows that patients infected with carbapenem‐resistant strains can have a mortality that reaches up to 50%, while mortality related to infections by sensitive strains is considerably lower [18, 21, 22, 26]. This discrepancy highlights the severity of the impact of AMR on patients’ health and emphasizes the urgency of effective interventions to control the spread of resistant strains.

In addition, clinical complications associated with infections by resistant *K. pneumoniae* are more frequent, resulting in prolonged hospitalizations and increased demand for intensive care. The treatment of these infections, often hampered by resistance to multiple classes of antibiotics, can lead to unfavorable outcomes, such as secondary infections and sepsis. Thus, resistance becomes not only an individual health issue, but a serious public health problem that requires immediate attention [41, 42].

The comparison of clinical outcomes between resistant and sensitive infections reveals that when sensitive *K. pneumoniae* infections are treated with appropriate antibiotics, the results tend to be more favorable. This underlines the importance of rapid and accurate identification of bacterial strains involved in infections, allowing the choice of appropriate treatments and, consequently, improving clinical outcomes [18, 26, 43]. These findings reinforce the need for diagnostic stewardship and investment in rapid diagnostic technologies to guide early targeted therapy.

#### 3.3.2. Treatment Options

Treatment options for resistant *K. pneumoniae* infections are limited, presenting a significant challenge for health professionals. Resistance to carbapenems in particular severely restricts antimicrobial choices, often resulting in less effective and potentially more toxic treatments [27, 43]. The use of alternative antibiotics, such as colistin and polymyxin B, although effective in some cases, is associated with a high risk of renal toxicity and the possibility of developing additional resistance ([44]).

A unique perspective of this review is the exploration of alternative anti‐virulence strategies, particularly natural adjuvants. Recent experimental evidence—including studies previously conducted by our research group [29]—highlights the potential of phenolic acids, specifically caffeic and trans‐ferulic acids. These compounds exert potent antimicrobial effects and, crucially, can inhibit up to 90% of biofilm formation in MDR/XDR *K. pneumoniae* isolates. This approach offers a vital complementary strategy to restore clinical efficacy when conventional carbapenems and aminoglycosides are rendered ineffective by enzymatic degradation.

In addition, alternative therapeutic approaches, such as the combination of different antibiotics, have shown variable results and do not always guarantee the desired effectiveness. Although combined therapy can increase the probability of treatment success, it can also increase the risk of adverse effects and complications ([43]; LEE et al., 2023; [1]). The absence of well‐defined guidelines for the management of multi‐resistant infections makes clinical practice even more challenging, requiring a careful and individualized evaluation of each case.

Given this panorama, the need for new treatment options and the development of innovative antimicrobial therapies is urgent. Beyond traditional antibiotics, promising alternatives are advancing through clinical development. Phage therapy has demonstrated success in compassionate‐use cases for carbapenem‐resistant *K. pneumoniae* infections, while monoclonal antibodies targeting specific virulence factors are entering early‐phase clinical trials. Additionally, novel *β*‐lactam/*β*‐lactamase inhibitor combinations, such as cefepime‐taniborbactam, have shown efficacy in phase III trials against KPC‐producing strains [1, 43]. These innovations are crucial to ensure that health professionals have effective tools to combat infections caused by resistant *K. pneumoniae*.

### 3.4. Impact on Public Health

#### 3.4.1. Epidemiology of Resistance

The growing AMR in *K. pneumoniae* has generated serious concerns among public health professionals. In recent decades, there has been an alarming increase in the prevalence of strains resistant to multiple classes of antibiotics, particularly in hospital environments. This phenomenon is often related to inadequate prescription practices and excessive use of antimicrobials, favoring the selection of resistant strains ([17]). The repercussions for public health are profound, since resistance in *K. pneumoniae* can lead to more severe infections, prolonged hospitalizations and increased mortality, configuring itself as a critical challenge for health systems [2, 18].

Epidemiological surveillance plays a vital role in understanding the dynamics of AMR. The collection and systematic analysis of data on the prevalence of resistant *K. pneumoniae* make it possible to identify resistance patterns, track outbreaks, and evaluate the effectiveness of public health interventions. Molecular epidemiology studies have been crucial for understanding the dissemination of high‐risk clones and resistance mechanisms in healthcare settings [6, 24]. The absence of accurate and updated data can result in inadequate interventions and aggravate the problem of resistance, underlining the urgent need for robust surveillance systems [21, 22, 27].

In addition, the epidemiological approach of AMR should be collaborative and adopt a One Health perspective, involving not only health authorities, research institutions and health professionals, but also integrating animal health and environmental sectors. The implementation of active surveillance programs must consider the interconnectedness of human, animal, and environmental reservoirs, as *K. pneumoniae* can circulate between these compartments, facilitating the spread of resistance genes [20, 45]. The promotion of information exchange between different regions and countries, coupled with intersectoral collaboration, is crucial to effectively combat resistance. Only through this coordinated One Health effort will it be possible to reduce the burden of AMR and protect public health.

#### 3.4.2. Infection Controls

The implementation of effective measures to control nosocomial infections is essential to prevent the spread of resistant *K. pneumoniae* in healthcare environments. Strict hygiene practices, such as the proper disinfection of surfaces and equipment, the correct use of personal protective equipment (PPE) and adherence to hand washing protocols, are essential to limit the spread of resistant pathogens. Studies show that the consistent application of infection control measures can significantly reduce the rate of hospital infections, contributing to the protection of vulnerable patients and reducing AMR [1, 9].

In addition to direct practices, education, and awareness about AMR are equally important. Health professionals should be trained about the importance of the rational use of antibiotics and infection control practices to prevent the spread of resistant strains [19, 26]. Education programs aimed at raising awareness among patients and the general population are crucial to promotes the responsible use of antimicrobials and reduces the self‐medication, a factor that contributes to increased resistance [12].

Finally, the implementation of infection control and education strategies should be accompanied by a continuous commitment to research and innovation. The development of new technologies, such as more effective disinfectants and real‐time monitoring systems, can complement traditional control measures [27, 46]. The integration of educational, practical, and research efforts is essential to address AMR in *K. pneumoniae* and ensure patient safety in healthcare environments.

### 3.5. Strategies to Mitigate the Spread of Resistance

#### 3.5.1. Rational Use of Antibiotics

The rational use of antibiotics is a fundamental strategy to mitigate the spread of AMR, especially in infections caused by *K. pneumoniae*. Proper prescription and careful use of antibiotics are essential to avoid the selection of resistant strains. The promotion of clinical guidelines and antibiotic therapy protocols that guide health professionals in the appropriate choice of antimicrobials is an effective approach to ensure that antibiotics are used only when necessary and in the correct dosages. Adherence to these guidelines can significantly reduce the incidence of resistance, contributing to the preservation of the effectiveness of existing antibiotics [27, 47].

In addition, the implementation of prescription review programs in hospitals, known as stewardship, has proven effective in promoting the rational use of antibiotics. These programs involve the continuous evaluation of antimicrobial therapies, aiming to adjust or discontinue treatment when appropriate. The involvement of pharmacists and infection control specialists in the review of prescriptions can result in a reduction in the duration of treatment and the use of narrower spectrum antibiotics, minimizing the impact on the patient’s microbiota and resistance selection [1, 19].

Therefore, the rational use of antibiotics should be a priority in resistance mitigation strategies. The continuous education of health professionals on the importance of proper prescription and the implementation of strict protocols is essential to combat AMR and ensure the effectiveness of available treatments [26, 27].

#### 3.5.2. Surveillance and Monitoring

Surveillance and monitoring of AMR are crucial components in the fight against the spread of resistant *K. pneumoniae*. Effective surveillance systems allow tracking the prevalence of resistant strains, identifying outbreaks, and evaluating the impact of the implemented interventions. The collection and analysis of data on resistance not only informs public health policies, but also provides valuable information for clinical practice, allowing the early identification of resistant strains and the adaptation of treatment approaches [6, 24].

Successful initiatives in different countries have demonstrated the effectiveness of surveillance in reducing AMR. For example, national surveillance programs, such as the National Antimicrobial Resistance Surveillance System in the United States and the Antimicrobial Resistance Surveillance Network in Europe, have contributed to the collection of comprehensive data on resistance. These systems have made it possible to compare trends over time and identify geographical areas with high resistance rates, directing control and prevention efforts [5, 48].

International collaboration is also essential to strengthen the surveillance and monitoring of these resistances. The exchange of information and experiences between countries and health organizations can lead to best practices and the development of common guidelines, increasing the effectiveness of interventions. The integration of genomic surveillance through WGS has further enhanced global tracking capabilities, enabling real‐time monitoring of resistance gene dissemination across borders [9, 11]. Thus, continuous surveillance and international collaboration are essential to combat resistance and protect public health.

#### 3.5.3. Education and Awareness

Education programs for health professionals are essential to address AMR effectively. Continuous training about the resistance mechanisms, the importance of the rational use of antibiotics and treatment guidelines can improve the understanding of professionals and, consequently, their prescription practices. These programs should include regular updates on local and global resistance trends, ensuring that professionals are aware of best practices and available treatment options [26, 49].

In addition, public awareness of the proper use of antibiotics is a crucial strategy to mitigate resistance. Public education campaigns that emphasize the importance of completing prescribed antibiotic courses and avoiding self‐medication are essential to reduce the selective pressure that contributes to the development of resistant strains. The promotion of responsible behaviors in relation to the use of antibiotics not only protects individuals but also contributes to the health of the complete community [12, 47].

The combination of education programs for health professionals and public awareness campaigns is essential to create an environment where the rational use of antibiotics is the norm. This integrated approach can lead to a significant reduction in AMR and improve clinical outcomes in infections by *K. pneumoniae* and other bacteria [1, 19].

#### 3.5.4. Research Innovations

The development of new antibiotics and therapeutic alternatives is a crucial priority in the fight against AMR. As *K. pneumoniae* strains become resistant to multiple classes of antibiotics, the need for new antimicrobial agents that can overcome this resistance becomes increasingly urgent. Recent advances include novel metallo‐*β*‐lactamase inhibitors such as ANT2681, which restores meropenem activity against NDM‐positive Enterobacterales, and siderophore‐antibiotic conjugates that exploit bacterial iron uptake systems, showing promising results against carbapenem‐resistant strains [44, 50]. Research on new antimicrobial molecules, combined with resistance modulation strategies, offers hope to recover the effectiveness of existing treatments and to develop new therapeutic options [28, 51].

Parallel to the development of new antimicrobial agents, research on alternative approaches targeting bacterial virulence mechanisms has gained significant attention. In this innovative landscape, Cavalcanti de Sá et al. [29] demonstrated that phenolic acids exhibit potent antimicrobial effects and effectively inhibit biofilm formation in MDR *K. pneumoniae* clinical isolates from a Brazilian public hospital, highlighting the potential of natural compounds as complementary therapeutic strategies.

Additionally, research in vaccines and immunotherapy has demonstrated promising potential. Several monoclonal antibody candidates targeting prevalent *K. pneumoniae* serotypes have entered early‐phase development, showing broad cross‐protection against ST258 carbapenem‐resistant strains, while phage therapy has emerged as a viable alternative against extensively drug‐resistant *K. pneumoniae* infections [37, 52, 53]. Immunotherapy approaches, including monoclonal antibodies and phage‐based therapies, represent innovative alternatives for managing resistant infections [35, 54].

Therefore, innovation in research is essential to face the challenge of AMR. Collaboration between researchers, pharmaceutical industries and academic institutions is essential to accelerate the development of new therapies, particularly through public‐private partnerships like CARB‐X that can bridge the funding gap in antibiotic development and create sustainable pipelines for novel antimicrobial agents [55, 56]. Investment in research and development is a critical component in the fight against resistance to *K. pneumoniae* and other resistant pathogens [12, 47].

#### 3.5.5. Multisectoral Collaboration

Collaboration between health professionals, researchers and public health authorities is essential to address AMR effectively. Resistance is not only a medical problem, but also a challenge involving economic, social, and environmental issues. The formation of multisectoral partnerships allows a comprehensive approach that considers all aspects of AMR, facilitating the exchange of information, resources, and best practices between different sectors [45, 57].

Examples of global partnerships and initiatives, such as WHO’s Global Action against AMR, demonstrate the potential of collaborative efforts to combat resistance. These initiatives aim to promote awareness of AMR, share data and experiences, and develop common guidelines and policies that can be implemented in different countries. Collaboration between countries, non‐governmental organizations and the scientific community is essential to meet the growing challenge of AMR at the global level [5, 15].

In addition, the inclusion of sectors beyond health, such as agriculture and industry, in the discussion on AMR is fundamental. The interaction between these sectors can contribute to the development of more effective strategies to control resistance, such as reducing the use of antibiotics in agricultural and livestock practices. Thus, an integrated approach involving multiple sectors is essential to mitigate the spread of resistance and protect public health [12, 47].

## 4. Final Consideration

AMR in *K. pneumoniae* represents a critical and multifaceted challenge to global public health, negatively impacting clinical practice and healthcare systems worldwide. This review has highlighted the alarming prevalence of co‐resistance to aminoglycosides and carbapenems, the complex underlying mechanisms, and the significant adverse effects on mortality and morbidity rates, as well as treatment costs. To effectively mitigate this resistance, a comprehensive approach is imperative, encompassing the rational use of antibiotics, robust epidemiological surveillance, and continuous education on AMR, complemented by research innovation through the development of new antibiotics, vaccines, and alternative therapies. Multisectoral collaboration, involving healthcare professionals, researchers, public health authorities, agriculture, and industry, is essential for effective resistance control within a One Health framework. Coordinated and integrated actions at local, national, and global levels are imperative to protect public health and ensure effective treatments for future generations. Addressing AMR in *K. pneumoniae* requires a collaborative, continuous, and multidisciplinary approach in research, education, and public health practices, ensuring that advances in medicine are not compromised by this growing problem.

## Author Contributions

Conceptualization: Rafael Artur de Queiroz Cavalcanti de Sá and Tainara Fernandes Dantas; Investigation: Sérgio Costa Júnior, Karolayne Oliveira da Silva and Maria Cecília Ferreira Galindo; Methodology: Amanda Vieira de Barros, John Eversong Lucena de Vasconcelos, Grażyna Kowalska and Rafal Rowinski; Software, Supervison and Final Draft: Radosław Kowalski, Henrique Douglas Melo Coutinho and Maria Betânia Melo Oliveira.

## Funding

No funding was received for this manuscript.

## Conflicts of Interest

The authors declare no conflicts of interest.

## Data Availability

The data that support the findings of this study are available from the corresponding author upon reasonable request.

## References

[1] Bassetti M. , Righi E. , Carnelutti A. , Graziano G. , Russo A. , Gremese I. , and Giacobbe D. R. , Nutritional support in patients with extracorporeal life support and ventricular assist devices, Current Opinion in Critical Care. (2018) 24, no. 4, 269–276, 10.1097/MCC.0000000000000512, 2-s2.0-85050194207. 29847341

[2] Codjoe F. S. and Donkor E. S. , Carbapenem Resistance: A Review, Medical Sciences. (2018) 6, no. 1, 10.3390/medsci6010001, 29267233. PMC5872158 29267233

[3] World Health Organization , WHO Bacterial Priority Pathogens List, 2024: Bacterial Pathogens of Public Health Importance to Guide Research, Development and Strategies to Prevent and Control Antimicrobial Resistance, 2024, World Health Organization.

[4] Arcari G. and Carattoli A. , Global Spread and Evolutionary Convergence of Multidrug-Resistant and Hypervirulent Klebsiella pneumoniae High-Risk Clones, Pathogens and Global Health. (2023) 117, no. 4, 328–341, 10.1080/20477724.2022.2121362, 36089853. 36089853 PMC10177687

[5] ECDC/EMEA , The Bacterial Challenge: Time to React, 2017, European Centre for Disease Prevention and Control, Disponível em: https://ecdc.europa.eu/en/publications-data/bacterial-challenge-time-react. Acesso em: 4 ago. 2024.

[6] Lee H. , Hong K. J. , Kim S. H. , Choi S. W. , and Shin J. H. , Increasing Prevalence of Carbapenem-Resistant *Klebsiella pneumoniae* in South Korea: A Nationwide Surveillance Study, Antimicrobial Resistance & Infection Control. (2020) 9, 10.1186/s13756-019-0590-0.

[7] Logan L. K. and Weinstein R. A. , The Epidemiology of Carbapenem-Resistant Enterobacteriaceae: The Impact and Evolution of a Global Menace, Journal of Infectious Diseases. (2017) 215, no. Supplement_1, S28–S36, 10.1093/infdis/jiw282, 2-s2.0-85020374494, 28375512. 28375512 PMC5853342

[8] Wang Q. , Wang X. , Wang J. , Ouyang P. , Jin C. , Wang R. , Zhang Y. , Jin L. , Chen H. , Wang Z. , Zhang F. , Cao B. , Xie L. , Liao K. , Gu B. , Yang C. , Liu Z. , Ma X. , and Wang H. , Phenotypic and Genotypic Characterization of Carbapenem-resistant Enterobacteriaceae: Data From a Longitudinal Large-Scale CRE Study in China (2012-2016), Clinical Infectious Diseases: An Official Publication Of The Infectious Diseases Society Of America. (2018) 67, no. Supplement_2, S196–S205, 10.1093/cid/ciy660, 2-s2.0-85056521952. 30423057

[9] Namy E. , Shah S. , Kim J. E. , Perez R. , and Bassler B. L. , An Autoinducer-Independent Rhlr Quorum-Sensing Receptor Enables Analysis Of Rhlr Regulation, PLoS Pathogens. (2019) 15, no. 6, e1007820, 10.1371/journal.ppat.1007820, 2-s2.0-85068116149, 31194839. 31194839 PMC6564026

[10] Keshtkaran A. , King S. J. , van Putten J. P. , and Wösten M. , Influence of Protein Glycosylation on Campylobacter fetus *Physiology* , Frontiers in Microbiology. (2020) 11, 10.3389/fmicb.2020.01191, 32625174. PMC7313396 32625174

[11] de Lima J. A. , de Souza Pinheiro L. , de Oliveira S. S. , Santos S. S. , de Castro C. N. , Gurgel-Gonçalves R. , and Cuba C. A. C. , First Report of Panstrongylus lignarius (Walker, 1873) (Hemiptera: Reduviidae: Triatominae) in the State of Acre, *Brazil* , Revista da Sociedade Brasileira de Medicina Tropical. (2019) 52, e20180307, 10.1590/0037-8682-0307-2018, 2-s2.0-85063276841. 30892400

[12] Ferreiro J. , Pando M. , Canas L. , Lodeiro M. , Gulin S. , and Loureiro A. , 2261. Impact of Antimicrobial Stewardship Program on Klebsiella pneumoniae and Escherichia coli Antimicrobial *Resistance* , Infectious Diseases. (2023) 10, no. Supplement_2, ofad500.1883, 10.1093/ofid/ofad500.1883.

[13] Agência Nacional de Vigilância Sanitária , Boletins e Relatórios Das Notificações de IRAS e Outros Eventos Adversos, 2024, ANVISA, Disponível em: https://www.gov.br/anvisa/pt-br/centraisdeconteudo/publicacoes/servicosdesaude/boletins-e-relatorios-das-notificacoes-de-iras-e-outros-eventos-adversos-1/boletins-e-relatorios-das-notificacoes-de-iras-e-outros-eventos-adversos.

[14] Pan-Americana da Saúde , Rede de Laboratórios de Vigilância da Resistência Antimicrobiana (RELAVRA): Dados e relatórios, 2022, OPAS/OMS, Disponível em: https://www.paho.org/en/amr-portal.

[15] WHO , Global Antimicrobial Resistance and Use Surveillance System (GLASS) Report, 2021, Disponível em: https://www.who.int/publications/i/item/9789240061700. Acesso em: 4 ago. 2024.

[16] Franklin A. M. , Weller D. L. , Durso L. M. , Bagley M. , Davis B. C. , Frye J. G. , Grim C. J. , Ibekwe A. M. , Jahne M. A. , Keely S. P. , Kraft A. L. , McConn B. R. , Mitchell R. M. , Ottesen A. R. , Sharma M. , Strain E. A. , Tadesse D. A. , Tate H. , Wells J. E. , Williams C. F. , Cook K. L. , Kabera C. , McDermott P. F. , and Garland J. L. , A One Health Approach for Monitoring Antimicrobial Resistance: Developing a National Freshwater Pilot Effort, Frontiers in Water. (2024) 6, 1359109, 10.3389/frwa.2024.1359109, 38855419. PMC11157689 38855419

[17] Velazquez-Meza M. E. , Galarde-López M. , Carrillo-Quiróz B. , and Alpuche-Aranda C. M. , Antimicrobial Resistance: One Health Approach, Veterinary World. (2022) 15, no. 3, 743–749, 10.14202/vetworld.2022.743-749, 35497962. 35497962 PMC9047147

[18] Perez F. , Hujer A. M. , Hujer K. M. , Decker B. K. , Rather P. N. , and Bonomo R. A. , Global Epidemiology of *Klebsiella pneumoniae* Infections: Prevalence and Resistance Patterns, Clinical Microbiology Reviews. (2018) 31, no. 2, e00028-17, 10.1128/CMR.00028-17.

[19] Kurtz J. , Simner J. M. , and Smith K. P. , Emergence of Multidrug-Resistant *Klebsiella pneumoniae*: A Review of the Current Landscape, Journal of Clinical Microbiology. (2019) 57, no. 3, e01526-18, 10.1128/JCM.01526-18.

[20] Asghar A. H. and Ali F. , Current Trends and Challenges in Antibiotic Resistance in *Klebsiella pneumoniae*: A Review, Current Microbiology. (2021) 78, no. 4, 1220–1230, 10.1007/s00284-021-02574-8.

[21] Chen T. , Lu C. , Zhang J. , Wang M. , Huang Y. , Wen F. , and Wang H. , The Epidemiology and Characteristics of Carbapenem-Resistant *Klebsiella pneumoniae* in a Tertiary Hospital, BMC Infectious Diseases. (2018) 18, no. 1, 1–10, 10.1186/s12879-018-3392-y.29291713

[22] Chen Y. , Jiang S. , Zheng J. , Zhu C. , Feng B. , Zhang Y. , and Zhang X. , The Prevalence of Aminoglycoside and Carbapenem Resistance Genes Among *Klebsiella pneumoniae* Clinical Isolates, BMC Infectious Diseases. (2018) 18, 10.1186/s12879-018-3224-8.

[23] Kamali F. , Hashemi A. , and Hamzehloie G. , Resistance Patterns and Molecular Mechanisms of *Klebsiella pneumoniae* in a Hospital Setting: A Review, Future Microbiology. (2020) 15, no. 4, 391–409, 10.2217/fmb-2019-0251.

[24] Oliveira M. J. , Silva C. A. , and Santos J. R. , The Molecular Epidemiology of *Klebsiella pneumoniae*: Implications for Infection Control, Revista da Sociedade Brasileira de Medicina Tropical. (2020) 53, e20190444, 10.1590/0037-8682-0444-2019.

[25] Pereira J. L. , Volcão L. M. , Klafke G. B. , Vieira R. S. , Gonçalves C. V. , Ramis I. B. , Rossetti M. L. R. , Pinto L. S. , and von Groll A. , Antimicrobial Resistance and Molecular Characterization of Extended-Spectrum *β*-Lactamases of Escherichia coli and Klebsiella spp. Isolates From Urinary Tract Infections in Southern Brazil, Microbial Drug Resistance. (2019) 25, no. 2, 173–181, 10.1089/mdr.2018.0046, 2-s2.0-85062765557, 30133334. 30133334 PMC6441284

[26] Martin M. J. , Corey B. W. , Sannio F. , Hall L. R. , Macdonald U. , Jones B. T. , Mills M. S. , Kwak Y. , Waterman E. , Bennett S. , Woodworth P. S. , Paolino G. S. , Russo A. , Luzzaro F. , Rossolini G. , and Docquier J. D. , Anatomy of an Extensively drug-resistant *Klebsiella pneumoniae* outbreak in Tuscany, Italy, Proceedings of the National Academy of Sciences. (2021) 118, no. 48, e2110227118, 10.1073/pnas.2110227118. PMC8640832 34819373

[27] Sharma P. , Pant S. , Rai S. , Yadav R. B. , and Sharma S. , Synthesis *of* silver nanoparticles using gum Arabic: Evaluation of its inhibitory action on Streptococcus mutans causing dental caries and endocarditis, Journal of Infection and Public Health. (2021) 14, no. 3, 324–330, 10.1016/j.jiph.2020.12.016, 33618277. 33618277 PMC7895472

[28] Bi M. , Mast Y. , Heipieper H. J. , Trost M. , Hatzinikolaou D. G. , Tsilia S. V. , Melissis V. S. , Vlachos M. G. , Typas V. , Papandreou N. C. , Ieropoulos Y. J. , Paraskevas S. J. , Grütter M. G. , Walsh F. , Kaasch A. J. , and Seifert H. , Clinical and Biological Features of Cutibacterium (Formerly Propionibacterium) avidum, an Underrecognized Microorganism, Clinical Microbiology Reviews. (2018) 31, no. 3, e00064-17, 10.1128/CMR.00064-17, 2-s2.0-85047912310, 29848774. 29848774 PMC6056840

[29] de Queiroz Cavalcanti de Sa R. A. , de Azevedo Ramos B. , de Caldas Padilha F. F. , Dantas T. F. , de Barros A. V. , de Veras B. O. , de Oliveira M. B. M. , and dos Santos Correia M. T. , Antimicrobial Effect and Inhibition of Biofilm Formation by Phenolic Acids on Multi-Drug Resistant Klebsiella pneumoniae Isolates From a Public Hospital From Pernambuco, Brazil, Evidência. (2024) 24, 10.18593/evid.34023.

[30] Al-Hassan L. , de Lima K. S. S. , El-Sowayel T. S. , Burgess J. S. , MacKenzie A. J. M. , Golovtsov I. V. , Scott J. S. , Owen D. J. , Foster S. J. , Rogers T. , Gillespie S. , Zloh M. , Taylor C. J. , Mast Y. , Heipieper H. J. , Trost M. , Hatzinikolaou D. G. , Tsilia S. V. , Melissis V. S. , Vlachos M. G. , Typas V. , Papandreou N. C. , Ieropoulos Y. J. , Paraskevas S. J. , Grütter M. G. , and Walsh F. , SC5005 dissipates the membrane potential to killStaphylococcus aureuspersisters without detectable Resistance, Journal of Antimicrobial Chemotherapy. (2021) 76, no. 8, 2049–2056, 10.1093/jac/dkab114. 33855344

[31] Ismail A. , Alzahrani H. S. , and El-Shaer J. M. A. , Molecular Characterization of Carbapenem-Resistant *Klebsiella pneumoniae* From a Teaching Hospital, Antimicrobial Resistance & Infection Control. (2019) 8, 10.1186/s13756-019-0492-3.

[32] Muller A. and Knieser S. J. , Antimicrobial Resistance in *Klebsiella pneumoniae*: The Importance of Surveillance and Control Measures, Infectious Disease Reports. (2020) 12, no. 1, 10.4081/idr.2020.8506.

[33] Dayan A. and Soria C. , *Klebsiella pneumoniae*: Characteristics, Resistance Mechanisms and Clinical Impact, Future Microbiology. (2019) 14, 387–397, 10.2217/fmb-2018-0183.

[34] Haque A. and Zhang R. , High Tuberculosis Burden Among Hiv-Infected Populations In *Thailand* Due To A Low-Sensitivity Tuberculin Skin Test, Journal of Infection and Public Health. (2020) 13, no. 4, 657–660, 10.1016/j.jiph.2019.08.010, 2-s2.0-85072581710. 31563472

[35] Shireen A. , Singh S. , and Kumar R. , Insights Into the Epidemiology and Resistance Mechanisms of *Klebsiella pneumoniae*: A Systematic Review, Infection and Drug Resistance. (2020) 13, 2829–2844, 10.2147/IDR.S273747.32884306

[36] Zhang X. and Pirofski L. A. , The Damage Response Framework And Infection Prevention: From Concept To Bedside, Infection Control & Hospital Epidemiology. (2020) 41, no. 3, 337–341, 10.1017/ice.2019.354. 31915082

[37] Bris J. , Chen N. , Supandy A. , Rendueles O. , and Van Tyne D. , Phage Therapy for *Klebsiella pneumoniae*: Understanding Bacteria–Phage Interactions for Therapeutic Innovations, PLOS Pathogens. (2025) 21, no. 4, e1012971, 10.1371/journal.ppat.1012971, 40198880. 40198880 PMC11978313

[38] Yu Y. and Al-Mugheed M. H. , Using Social Network Tools To Facilitate Cultural Adjustment Of Self-Initiated Malaysian Female Expatriate Nurses In *Saudi* Arabia, Journal of Infection and Public Health. (2021) 14, no. 3, 380–384, 10.1016/j.jiph.2020.11.008. 33621801

[39] Kumari L. S. , Rajapakse R. P. V. J. , and Senanayake S. S. H. M. L. , Rapid Whole Genome Sequencing for AMR Surveillance in Low- and Middle-Income Countries: Oxford Nanopore Technology Reveals Multidrug-Resistant *Enterobacter cloacae* Complex From Dairy Farms in Sri Lanka, BMC Veterinary Research. (2025) 21, no. 1, 10.1186/s12917-025-04800-1, 40382559. PMC12084958 40382559

[40] Strike W. , Kato J. B. , Nabadda S. L. , and Joloba M. R. , Implementing Wastewater-Based Epidemiology for Long-Read Metagenomic Sequencing of Antimicrobial Resistance in Kampala, Uganda, Microorganisms. (2025) 13, no. 6, 10.3390/microorganisms13061240, 40572128. PMC12195534 40572128

[41] Mendes G. , Silva C. R. V. , Ramos C. , Duarte S. S. , and Silva L. P. , Virulence Factors in Carbapenem-Resistant Hypervirulent *Klebsiella pneumoniae* , Frontiers in Microbiology. (2023) 14, 1325077, 10.3389/fmicb.2023.1325077, 38098668. 38098668 PMC10720631

[42] Salazar R. , Esparza R. , and Ruiz M. , Carbapenem-Resistant *Klebsiella pneumoniae*: A Study of Risk Factors and Clinical Outcomes, BMC Infectious Diseases. (2020) 20, no. 1, 10.1186/s12879-020-4818-x.

[43] Nisanian S. , Souli M. , and Koratzanis P. , The Impact of Resistance to Aminoglycosides on Treatment Outcomes in *Klebsiella pneumoniae* Infections, BMC Infectious Diseases. (2020) 20, no. 1, 10.1186/s12879-020-04967-5.

[44] Zalacain M. , Lozano C. , Llanos A. , Sprynski N. , Valmont T. , De Piano C. , Davies D. , Leiris S. , Sable C. , Ledoux A. , Morrissey I. , Lemonnier M. , and Everett M. , Novel Specific Metallo-*β*-Lactamase Inhibitor ANT2681 Restores Meropenem Activity to Clinically Effective Levels Against NDM-PositiveEnterobacterales, Antimicrobial Agents and Chemotherapy. (2021) 65, no. 6, e00203-21, 10.1128/aac.00203-21. 33820763 PMC8315971

[45] OIE , Antimicrobial Resistance: A Global One Health Challenge, 2020, World Organization for Animal Health, Disponível em: https://www.oie.int/en/what-we-do/global-initiatives/antimicrobial-resistance/. Acesso em: 4 ago. 2024.

[46] Grisolia J. A. , Lopes S. C. P. , Lacerda M. V. G. , and Croda J. , Tuberculosis: Renewed Challenge *In* Brazil, Revista da Sociedade Brasileira de Medicina Tropical. (2018) 51, no. 1, e20180054, 10.1590/0037-8682-0054-2018, 2-s2.0-85043333140. 29513837

[47] Chavda K. D. and Melano R. G. , Incidence, Persistence And Clearance Of Cervical Human Papillomavirus Among Women In Guangdong, China 2007–2018: A Retrospective Cohort Study, Journal of Infection and Public Health. (2021) 14, no. 1, 42–49, 10.1016/j.jiph.2020.11.011. 33341483

[48] Centers for Disease Control and Prevention (CDC) , Antibiotic Resistance Threats in the United States, 2019, 2019, Department of Health and Human Services, Disponível em: https://www.cdc.gov/drugresistance/threat-report-2019.html. Acesso em: 4 ago. 2024.

[49] Qadir A. , Ahmed A. , and Mahmood H. , Prevalence and Risk Factors of Carbapenem-Resistant *Klebsiella pneumoniae* in Patients With Diabetes: A Multicenter Study, International Journal of Diabetes in Developing Countries. (2019) 39, no. 1, 52–58, 10.1007/s13410-018-0658-1.

[50] Reddy N. , Balieiro A. , Silva J. , Gouws C. , Mutshembele A. , Arvidsson P. , Kruger H. , Govender T. , and Naiker T. , Navigating the Complexities of Drug Development for Metallo-*β*-Lactamase Inhibitors, RSC Medicinal Chemistry. (2025) 16, no. 8, 3393–3415, 10.1039/d5md00035a, 40521342. 40521342 PMC12159872

[51] Cohen M. L. , The Threat of Antibiotic Resistance: A Call to Action, Nature Reviews Microbiology. (2020) 18, no. 4, 215–216, 10.1038/s41579-020-0350-8.

[52] Banerjee K. , Motley M. , Boniche-Alfaro C. , Bhattacharya S. , Shah R. , Ardizzone A. , and Fries B. , Patient-Derived Antibody Data Yields Development of Broadly Cross-Protective Monoclonal Antibody Against ST258 Carbapenem-Resistant *Klebsiella pneumoniae* , Microbiology Spectrum. (2022) 10, no. 4, e01760-22, 10.1128/spectrum.01760-22, 35862974. 35862974 PMC9430753

[53] Motley M. , Diago-Navarro E. , Banerjee K. , Inzerillo S. , and Fries B. C. , The Role of IgG Subclass in Antibody-Mediated Protection Against Carbapenem-Resistant *Klebsiella pneumoniae* , MBio. (2020) 11, no. 5, e02059-20, 10.1128/mbio.02059-20. 32900809 PMC7482069

[54] Reza F. , Khan M. S. J. S. , and Hasan M. J. , The Current Burden of Carbapenemases: Review *of* Significant Properties and Dissemination among Gram-Negative Bacteria, Antibiotics. (2020) 9, no. 4, 10.3390/antibiotics9040186, 32316342. PMC7235769 32316342

[55] Miethke M. , Pieroni M. , Weber T. , Brönstrup M. , Hammann P. , Halby L. , Arimondo S. , Glaser P. , Bazga B. , Winterhalter C. , Lebeke D. , and Müller R. , Towards the Sustainable Discovery and Development of New antibiotics, Chemistry. (2021) 5, no. 10, 726–749, 10.1038/s41570-021-00313-1, 34426795. PMC8374425 34426795

[56] Outterson K. , Rex J. H. , Jinks T. , Jackson P. , Hallinan J. , Karp S. , Hung D. , Franceschi F. , Merkeley T. , Houchens C. , Dixon D. , Kurilla M. , Aurigemma R. , and Larsen J. , Accelerating Global Innovation to Address Antibacterial Resistance: Introducing CARB-X, Nature Reviews Drug Discovery. (2016) 15, no. 9, 589–590, 10.1038/nrd.2016.155, 2-s2.0-84980002235, 27469032. 27469032

[57] WHO , Global Action Plan on Antimicrobial Resistance, 2015, World Health Organization, Disponível em: https://www.who.int/publications/i/item/global-action-plan-on-antimicrobial-resistance. Acesso em: 4 ago. 2024.

